# Evaluating the Efficacy of Massage Intervention for the Treatment of Poststroke Constipation: A Meta-Analysis

**DOI:** 10.1155/2020/8934751

**Published:** 2020-06-11

**Authors:** Qiu-Shuang Wang, Ya Liu, Xiang-Ni Zou, Yan-Ling Ma, Gen-Li Liu

**Affiliations:** ^1^The Second Affiliated Hospital of Harbin Medical University, Harbin, China; ^2^Heilongjiang University of Chinese Medicine, Harbin, China; ^3^Department of Interventional Radiology, The Third Affiliated Hospital of Harbin Medical University, Harbin, China; ^4^Acupuncture Wards, The Second Affiliated Hospital, Heilongjiang University of Chinese Medicine, Harbin, China

## Abstract

**Background:**

It is essential to determine a safe and effective method for treating constipation after stroke. Massage has been widely used in recent years. However, meta-analysis data on the efficacy of massage for the treatment of constipation experienced after stroke are almost nonexistent.

**Objective:**

This review aimed to examine the effectiveness of using massage therapy to treat constipation in patients who suffered a stroke event.

**Methods:**

This systematic review adhered to the reporting guidelines for Preferred Reporting Items for Systematic Reviews and Meta-Analyses. Electronic databases, including Cochrane Library, PubMed, EMBASE, Web of Science, China Biology Medicine, Wan Fang Data, VIP Database for Chinese Technical Periodicals, and National Knowledge Infrastructure, were searched for relevant studies on the efficacy of massage for the treatment of poststroke constipation. Rev-Man 5.3 software was used to analyze the study data.

**Results:**

A total of 11 randomized controlled trials with 1045 patients were included. A statistically significant difference in the total effective rates was found between the massage and control groups (OR = 4.96; 95% confidence interval (CI): 2.81, 8.76; *P* < 0.001). Compared with the control groups, the massage group had markedly reduced incidences of constipation (OR = 0.34; 95% CI: 0.25, 0.47; *P* < 0.001) and of four symptoms of discomfort (*P* < 0.001). The frequency of defecation on day two and day three in the massage group was significantly higher than that in the control group (*P* < 0.001).

**Conclusion:**

Our results strongly suggest that massage can effectively reduce the incidence and severity of constipation after stroke. However, large, multicenter, long-term, and high-quality randomized controlled trials need to be conducted to establish a definitive conclusion.

## 1. Introduction

Eleven studies have confirmed that constipation is one of the most common complications that occur after stroke [1]. Due to the influences of multiple factors, such as limited physical mobility, changes in the posture assumed during defecation due to stroke-related postural changes, adverse drug reactions, and emotional changes, patients after stroke have a significantly higher probability of having constipation than healthy people. Constipation not only affects patients' quality of life but also hinders their recovery process after stroke; it can even cause the recurrence of cerebrovascular diseases [2]. Presently, the treatment for constipation includes drug therapy and nondrug therapy. Drug therapy usually involves the use of stool softeners, prokinetic agents, and osmotic and stimulant laxatives. Unfortunately, these drugs usually have side effects such as bloating, nausea, and diarrhea [3]. In contrast, nondrug therapy includes dietary and lifestyle changes and other alternative therapies. Due to the shortcomings of the aforementioned therapeutic methods, it is vital to seek a treatment method that is effective and has few side effects. Massage is one such method and has been widely used in recent years [4, 5]. Abdominal massage, in particular, is a simple, convenient, and low-cost treatment method [6]. The principle of massage involves the use of a variety of techniques to activate the proper movement of the connective tissue and the superficial and deep muscle layers. Studies have confirmed that massage is effective in relieving muscle tension, reducing pain, facilitating the optimal functioning of the cardiovascular and nervous systems, and relieving constipation in children or the elderly [7, 8].

Presently, only a few meta-analyses are available on the effectiveness of massage intervention for the treatment of constipation after stroke. Therefore, the present meta-analysis aimed to systematically review and evaluate the effectiveness of massage for the treatment of constipation in patients after a stroke.

## 2. Materials and Methods

### 2.1. Design

Several databases, including the Cochrane Library, PubMed, EMBASE, and Web of Science, were searched for relevant English literature, whereas Chinese Biomedical Literature (CBM) database, Wan Fang database (Wan Fang), Database for Chinese Technical Periodicals (VIP), and China National Knowledge Infrastructure (CNKI) were searched for relevant Chinese literature. The Chinese and English electronic databases were searched for the literature added from the date of their establishment to May 2019.

The Chinese Clinical Trial Registration Center, Index to Scientific and Technical Proceedings, and Clinical Trials websites were also searched for relevant unpublished literature and clinical trials. There were no language or date restrictions on the retrieval of literature.

### 2.2. Search Strategy

#### 2.2.1. PubMed

The search terms used were as follows: “constipation” (MeSH), or “gastrointestinal constipation,” or “bowel disorder,” or “colonic inertia,” or “dyschezia”; “massage” (MeSH), or “oil massage,” or “therapeutic touch,” or “manual therapy,” or “massotherap^∗^,” or “friction,” or “tactile-kinesthetic”; “stroke” (MeSH), or “apoplexy,” or “cerebrovascular accident^∗^,” or “cerebrovascular,” or “apoplexy cerebrovascular stroke,” or “brain vascular accident,” or “cerebral stroke,” or “acute stroke,” or “acute cerebrovascular accident.” The complete search strategy is shown in the Supplementary Material (available here).

### 2.3. Inclusion Criteria

The inclusion criteria were as follows. (1) Participants had to be adults (over 18 years old) experiencing constipation after a first or recurrent stroke (we included adult patients with stroke irrespective of the stage of their disease (acute, subacute, or chronic)). (2) To reduce bias, we included only randomized controlled trials. (3) There were no language restrictions in the inclusion of studies. (4) The intervention groups in the included studies underwent only massage therapy. (5) The control groups received nonmassage therapy such as dietary and life guidance. (6) Outcome measures were total effective rate, incidence of constipation, time of first bowel movement, and symptoms of first bowel movement.

### 2.4. Exclusion Criteria

The following patients were excluded: (1) patients with severe heart, brain, and kidney complications; (2) patients who received other interventional measures such as moxibustion, ear acupuncture, and food therapy; (3) patients who underwent either an electric massage or a combined nursing intervention with abdominal massage; (4) patients with intestinal diseases; (5) patients with habitual constipation.

### 2.5. Data Extraction

Two evaluators screened the included literature independently according to the inclusion and exclusion criteria. First, they read the titles and abstracts for preliminary screening and excluded duplicates and obviously irrelevant articles. After cross-checking, the full texts of all possibly relevant articles were collected and carefully evaluated before the final list was made. The two authors independently extracted data from the included studies. In case of disagreement between the two evaluators, calibration exercises were performed before starting the review to ensure consistency between the decisions of the reviewers, and any dispute was resolved by third-party arbitration. The following information was extracted using predetermined data formats: author, publication date, intervention, sample size, intervention time, and outcome indicators. Literature quality evaluation was conducted independently according to the guidelines of the Cochrane Systematic Review Handbook [9]. The following six components reported in the literature were evaluated: (1) random sequence generation; (2) allocation concealment; (3) blinding; (4) incomplete outcome data and selective report; (5) comparability of baseline data; (6) intentional treatment.

### 2.6. Data Analysis

Rev-Man 5.3 statistical software provided by the Cochrane collaboration was used for statistical analysis. Heterogeneity among studies was tested using the chi-square test, and the significance level was set at *P*=0.10. If the significant heterogeneity was not high (*P* ≥ 0.1, *I*^2^ ≤ 50%), a fixed-effect model was selected. If the significant heterogeneity existed (*P* < 0.1, *I*^2^ > 50%), the source of the heterogeneity was analyzed. Sensitivity analysis was used to evaluate the stability of the analysis results. Funnel plot analysis was used to determine the existence of publication bias when the number of included studies exceeds ten according to the Cochrane manual. For dichotomous variables, odds ratio (OR) was adopted, and 95% confidence interval (CI) was calculated. The test level was set as *α* = 0.05. For articles with large differences in research content, method, or outcome evaluation index as compared with others, only descriptive analysis was performed.

## 3. Results

### 3.1. Included Studies and Their Basic Characteristics

**Figure 1:**
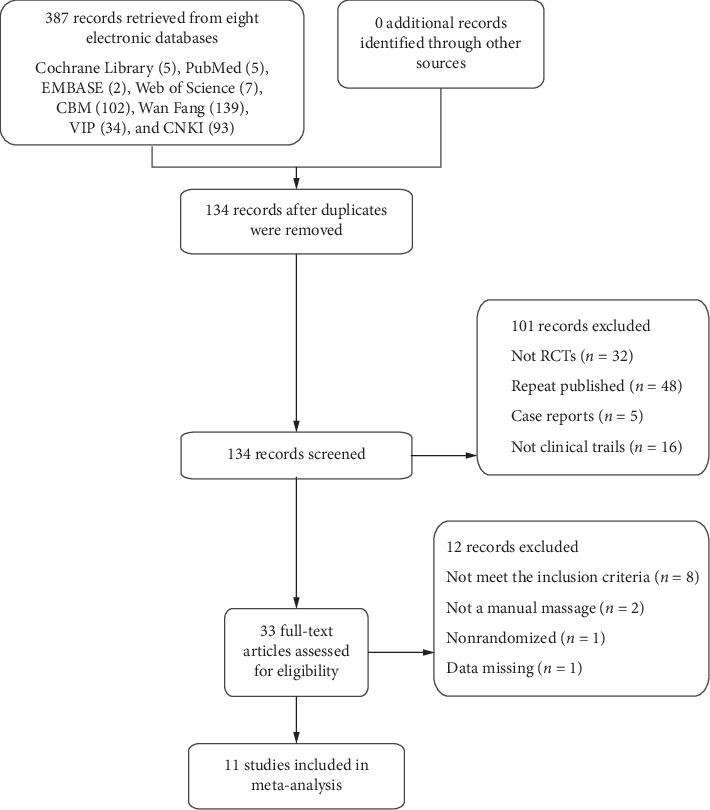
Flow chart of search results and study selection. RCT, randomized controlled trial.

First, 387 articles were selected from eight databases after preliminary screening. The search results were as follows: Cochrane Library, 5; PubMed, 5; EMBASE, 2; Web of Science, 8; CBM, 102; Wan Fang, 138; VIP, 34; CNKI, 93. After eliminating duplicate articles, the total number of selected articles was reduced to 134. After reading the titles and abstracts, case reports and other related papers such as reviews, statements, and authors' responses to reviews were removed; 43 articles were retained. Finally, the full texts of the remaining articles were evaluated and studies without control or randomization were excluded. Thus, 11 articles were finally included in this study for subsequent analysis. The specific search process and study selection is shown in Figure 1. A detailed description of the general data from the 11 randomized controlled trials with 1045 patients is shown in Table 1.

### 3.2. Methodological Quality of the Included Trials

**Figure 2:**
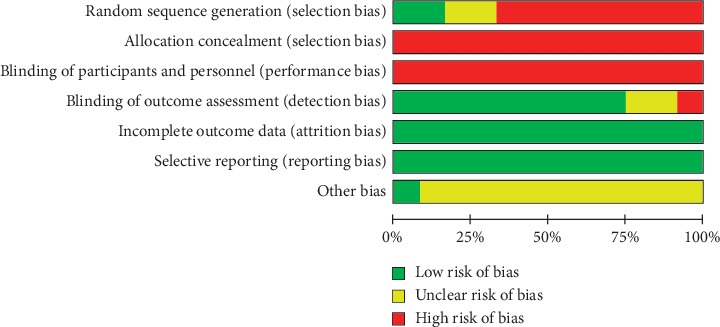
The methodological quality evaluation of bias risk.

All 11 included articles mentioned random allocation; however, only one article described the use of random allocation according to the random number table method [12]. No article described the strategy used for allocation blinding. Methodological quality evaluation of bias risk is shown in Figure 2.

### 3.3. Meta-Analysis Results

#### 3.3.1. Comparison of Total Effective Rate between Massage and Control Groups

**Figure 3:**
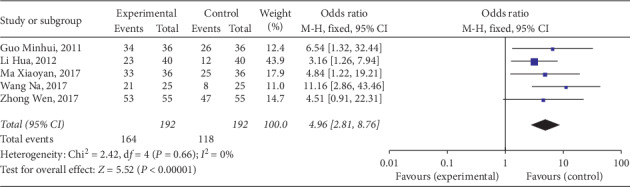
Forest plot of total effective rate. CI, confidence interval.

Total effective rate was evaluated in five trials. A total of 384 subjects were included in these studies [10, 13–15, 17]: 192 subjects were treated with massage and 192 were assigned to control groups. No significant heterogeneity was observed among these studies (*P*=0.66, *I*^2^ = 0%). Therefore, the fixed effects model was used for analysis. Compared with the control groups, symptoms of constipation in the massage groups demonstrated significant improvement (OR = 4.96; 95% CI: 2.81, 8.76; *P* < 0.001) (Figure 3).

#### 3.3.2. Comparison of the Incidences of Constipation after Stroke between the Massage and Control Groups

**Figure 4:**
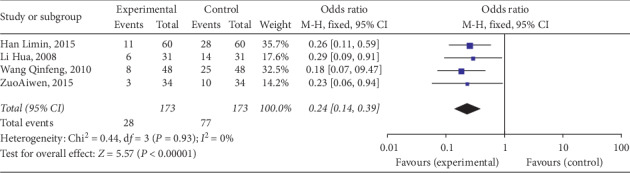
Forest plot of incidence of constipation. CI, confidence interval.

The incidence of constipation after stroke was evaluated in four trials [11, 18–20]. A total of 346 subjects were included in these studies: 173 were treated with massage and 173 were assigned to control groups. No significant heterogeneity was observed among these studies (*P*=0.93, *I*^2^ = 0%), and hence, the fixed effects model was used for analysis. Compared with the control groups, the massage groups had noticeably reduced incidences of constipation (OR = 0.24; 95% CI: 0.14, 0.39; *P* < 0.001) (Figure 4).

#### 3.3.3. Comparison of the Incidences of Defecation after Stroke between the Massage and Control Groups

Three studies reported the first defecation time [12, 16, 18]. However, one of the studies reported the time of the first bowel movement only in hours in both groups [18], while the other two articles reported it on a daily basis. For this reason, only those two articles were analyzed. The results showed that there was no statistically significant difference on the first day in the incidence of defecation between the massage and control groups. However, on the second and third days, the incidence of defecation in the massage group was significantly higher than that in the control group (the second day (31.88% and 16.13%, OR 2.41, 95% CI 1.22–4.77; *P* < 0.05) and the third day (35.00% and 15.48%, OR 3.00, 95% CI: 1.74–5.17; *P* < 0.05)) (Table 2).

#### 3.3.4. Comparison of Symptoms of First Bowel Movement of Constipation after Stroke between the Massage and Control Groups

Two studies [12, 16] reported the symptoms associated with the first bowel movement as follows: defecation difficulties, prolonged defecation time, dry stool, and endless defecation. The analysis results show that the rate of the above symptoms in the massage group was significantly lower than that in control group (*P* < 0.05) (Table 3).

### 3.4. Effects of Massage on Blood Pressure and Heart Rate

One study reported the changes in blood pressure and heart rate before and after intervention in the massage group, and the results showed no statistically significant difference (*P* > 0.05) [16].

### 3.5. Reports on Adverse Events

In this analysis, 11 articles were included: five articles mentioned that none of the adverse reactions were found in the massage therapy group [10, 11, 13–17, 19, 20], while the other articles did not mention the adverse reaction. However, so far, few adverse events related to massage have been reported in the literature, so it can be seen that massage is a relatively safe treatment method.

### 3.6. Sensitivity Analysis

Sensitivity analysis was used to evaluate the stability of the analysis of the results. The results of the fixed effects model and random effects model were consistent with each other, suggesting that the meta-analysis results were relatively stable (Table 4).

## 4. Discussion

### 4.1. Treatment Status of Constipation after Stroke

Constipation commonly occurs after a stroke [1]. Constipation can seriously damage the patients' health and affect the recovery of stroke patients [21].

At present, the treatment of constipation after stroke mainly includes using laxative, enema agents, and prokinetic drugs [21]; changing lifestyle [22–24]; dietary adjustments [25, 26]; using traditional Chinese herbal [27]; acupuncture [2]; and transcutaneous electrical acustimulation (TEA) [28]. However, stroke patients often show physical activity disorders and therefore lifestyle changes are difficult. Furthermore, the above drug treatment may be related to some adverse reactions such as abdominal distension, dehydration, and easy recurrence [25, 27]. Additionally, acupuncture and biofeedback therapy have high requirements on operators. Acupuncture points and techniques can affect the therapeutic effect [29, 30].

#### 4.1.1. Advantages of Massage in Poststroke Constipation

Massage is a technique to activate the deep and surface connective tissue and stromal cells; it has existed in ancient civilizations for thousands of years [31]. It can not only promote physical and intellectual development but also enhance immunity, promote digestion, and absorption [32]. Massage has the following advantages compared with the aforementioned treatments for constipation therapy after stroke. First, it is a simple and easy-to-learn procedure [33]; second, patients have no other discomforts leading to high compliance; third, there have been no reported side effects [34]; finally, it is a low-cost treatment, which can reduce medical costs and consequently reduce social burden [35]. Therefore, in practice, carrying out a massage treatment on patients with poststroke constipation is of great importance.

#### 4.1.2. Therapeutic Effect of Massage on Poststroke Constipation

This study suggests that massage intervention induced alleviation of poststroke constipation and reduced the incidence of constipation in patients with stroke. These findings are consistent with the results reported by Coggrave et al. [21]. In addition, massage can shorten the time of first defecate and improve discomfort associated with defecation. The possible reasons for these findings could be the following: first, abdominal massage promotes intestinal peristalsis; second, it improves the speed of gastrointestinal peristalsis, which in turn speeds up the discharge of gastrointestinal contents; third, it enhances the secretion of digestive secretions, which helps to reduce heavy intestinal absorption of moisture and in turn softens feces to aid easier discharge; finally, with regard to traditional Chinese medicine, massage therapy stimulates the vital energy of the human body and adjusts the balance of Yin and Yang, which in turn makes vital energy flow outside the pulse and prevents the invasion of diseases.

#### 4.1.3. Limitations of This Study

First, only 11 articles were included in this meta-analysis study. According to the Cochrane manual, the funnel plot is only applicable when the number of included studies exceeds ten. The number of included studies for each prognostic indicator in this study was less than eight. Therefore, funnel plot analysis could not be performed. Second, the analyzed studies included 11 controlled studies, all of which were randomized. However, only one study reported specific randomization methods. In addition, it is difficult to blind participants and personnel when patients are involved in massage. Blinding protocols were not mentioned in any of the studies. Third, although the literature retrieval was conducted in both Chinese and English, the research on the intervention of massage on constipation after stroke was only reported in Chinese literature; no English literature has been published on this subject yet. Fourth, although the incidence of stroke can be divided into acute and chronic stages, the present study did not define each stage of stroke, which may lead to increased risk of bias. Therefore, the stage of the stroke and patients' activities of daily living should be considered in future analysis.

### 4.2. Implications for Nursing Practice

Massage therapy for the treatment of poststroke constipation is effective and has been well confirmed in this study. However, more multicenter, large-sample, and randomized controlled trials need to be conducted to establish a standardized program for the treatment of poststroke constipation in China. Generally, constipation after stroke is ignored by patients and their families. Therefore, clinical nursing managers should work on establishing strategies and formulating nursing measures to prevent and treat constipation in patients with stroke.

### 4.3. Implications for Nursing Policy

This study shows that massage not only relieves but also prevents constipation after stroke. To reduce the pain experienced by patients and to accelerate their recovery, whether in hospitals or communities, nursing managers should improve the awareness of stroke patients and their families in relation to preventing constipation. This can be done through health guidance and by encouraging patients to perform self-massage. For in-patients with a history of constipation, nurses should perform standardized acupoint massage, and nursing managers should take the lead in formulating standardized massage interventions.

## 5. Conclusions

In summary, the current meta-analysis indicates that massage therapy is an effective and safe intervention for the treatment of constipation in patients with stroke. However, further long-term, high-quality studies are needed to confirm the long-term effect of massage on poststroke constipation.

## Figures and Tables

**Table 1:** Characteristics of the included studies.

First author, year	Inclusion and exclusion criteria	Type of study	Participant (I/C)	Interventions	Outcome
Zhong et al. [10]	Inclusion criteria: a, b, c; exclusion criteria: f, g, h	RCT	55/55	Meridian massage once a day for 14 consecutive days	①
Han [11]	Inclusion criteria: d, e; exclusion criteria: i, h	RCT	60/60	Abdominal massage twice a day for 14 consecutive days	②
Jiao and Zhai [12]	Inclusion criteria: b; exclusion criteria: i, j	RCT	80/75	Periumbilical massage and acupoint massage twice a day for 3 consecutive days	③④
Ma [13]	Inclusion criteria: b; exclusion criteria: i	RCT	36/36	Acupoint massage twice a day for 14 consecutive days	①
Wang [14]	Inclusion criteria: b, d; exclusion criteria: i	RCT	25/25	Acupoint massage once a day for 21 consecutive days	①
Li and Guo [15]	Inclusion criteria: b; exclusion criteria: i	RCT	40/40	Acupoint massage once a day for 7 consecutive days	①
Pan and Xin [16]	Inclusion criteria: b; exclusion criteria: i	RCT	80/80	Acupoint massage twice a day for 3 consecutive days	③④⑤
Guo et al. [17]	Inclusion criteria: b, d; exclusion criteria: i	RCT	36/36	Acupoint massage once a day for 3 consecutive days	①
Zuo [18]	Inclusion criteria: b, e; exclusion criteria: i	RCT	34/34	Acupoint massage once a day for 3 consecutive days	②③
Li et al. [19]	Inclusion criteria: b; exclusion criteria: i, l, m	RCT	31/31	Point rubbing, abdominal massage twice a day for 14 consecutive days	②
Wang and Yao [20]	Inclusion criteria: b; exclusion criteria: h, i	RCT	48/48	Acupoint massage twice a day for 10 consecutive days	②

Inclusion and exclusion criteria: a: candidates are aged between 55 and 85; b: diagnosis of cerebral apoplexy met the diagnostic criteria of the fourth national meeting of cerebrovascular diseases and was confirmed by CT or MRI; c: defecation frequency ≤ 3 times per week, weight of feces < 35 g or ≥ 25% per day; d: no dysphagia; e: no habitual constipation before admission; f: patients with mental retardation; g: epilepsy; h: other serious diseases; i: organic intestinal lesions; j: swallowing dysfunction; k: abstemious constipation; l: disturbance of consciousness; m: organic disease of anus. Outcome: ① total effective rate; ② incidence of constipation; ③ time of first bowel movement; ④ symptoms of first bowel movement; ⑤ blood pressure, heart rate comparison. I/C, intervention group/control group; RCT, randomized controlled trial.

**Table 2:** Meta-analysis of comparison of incidences of defecation after stroke between the massage and control groups.

Time of first bowel movement	Included studies	The total number of cases	Heterogeneity test	Analytical model	Incidence of massage group (%)	Incidence of control group (%)	*P*
First day	2	315	*I* ^2^ = 0%, *P*=0.91	Fixed effects model	11.25 (18/160)	14.19 (22/155)	0.43
Second day	2	315	*I* ^2^ = 36%, *P*=0.21	Fixed effects model	31.88 (51/160)	16.13 (25/155)	0.01
Third day	2	315	*I* ^2^ = 0%, *P*=0.54	Fixed effects model	35.00 (56/160)	15.48 (24/155)	<0.01

**Table 3:** Meta-analysis of the accompanying condition of first bowel movement in the two groups.

Accompanying condition of first bowel movement	Included studies	The total number of cases	Heterogeneity test	Analytical model	Incidence of massage group (%)	Incidence of control group (%)	*P*
Use of laxatives	2	315	*I* ^2^ = 81%, *P*=0.02	Random effects model	23.12 (37/160)	60.00 (93/155)	<0.01
Defecation difficulty	2	315	*I* ^2^ = 0%, *P*=0.87	Fixed effects model	42.50 (68160)	61.94 (96/155)	<0.01
Prolonged defecation	2	315	*I* ^2^ = 0%, *P*=1.00	Fixed effects model	48.13 (77/160)	66.45 (103/15)	<0.01
Dry stool	2	315	*I* ^2^ = 0%, *P*=0.99	Fixed effects model	35.63 (57/160)	59.35 (92/155)	<0.01
Endless defecation	2	315	*I* ^2^ = 0%, *P*=0.91	Fixed effects model	33.13 (53/160)	52.90 (82/155)	<0.01

**Table 4:** Sensitivity analysis results.

Evaluation index	Fixed effects model (OR (95% CI))	Random effects model (OR (95% CI))
Total effective rate	4.96 (2.81, 8.76)	4.90 (2.76, 8.71)
Incidence of constipation	0.24 (0.14, 0.39)	0.24 (0.14, 0.39)
Time of first bowel movement on the first day	0.77 (0.39, 1.49)	0.77 (0.39, 1.49)
Time of first bowel movement on the second day	2.43 (1.42, 4.17)	2.41 (1.22, 4.77)
Time of first bowel movement on the third day	2.99 (1.73, 5.17)	3.00 (1.74, 5.17)
Use of laxatives	0.18 (0.11, 0.30)	0.16 (0.05, 0.58)
Defecation difficulty	0.45 (0.29, 0.71)	0.45 (0.29, 0.73)
Prolonged defecation	0.47 (0.29, 0.73)	0.47 (0.29, 0.73)
Dry stool	0.38 (0.24, 0.59)	0.38 (0.24, 0.59)
Endless defecation	0.44 (0.28, 0.69)	0.44 (0.28, 0.69)

## Supplementary Materials

Supplementary Materials This review focuses on studies evaluating the effects of massage in poststroke constipation in comparison with other interventions. Research databases including PubMed, Database for Chinese Technical Periodicals, China National Knowledge Infrastructure, Web of Science, Cochrane library, EMBASE, Chinese Biomedical Literature Database and Wan fang Database were used to identify the related literature. We broke the review question into PICOS components: poststroke constipation (P), massage or abdominal massage (I), dietary guidelines or other nonmassage therapies (C), effective relief of constipation symptoms and associated symptoms (O), and randomized, controlled trials (RCTs) (S). According to PICOS components, we developed the searching strategy of this systematic review. The databases were searched with a combination of medical subject heading (MeSH) terms and text words for target population and interventions using massage. The detailed retrieval strategy is in the appendix file.
