# Supplementary material for: Evaluating the Efficacy of Massage Intervention for the Treatment of Poststroke Constipation: A Meta-Analysis
Source: Evid Based Complement Alternat Med. 2020 Jun 11;2020:8934751. doi: 10.1155/2020/8934751 (PMC7305530; doi:10.1155/2020/8934751)
Supplement: Supplementary Materials — This review focuses on studies evaluating the effects of massage in poststroke constipation in comparison with other interventions. Research databases including PubMed, Database for Chinese Technical Periodicals, China National Knowledge Infrastructure, Web of Science, Cochrane library, EMBASE, Chinese Biomedical Literature Database and Wan fang Database were used to identify the related literature. We broke the review question into PICOS components: poststroke constipation (P), massage or abdominal massage (I), dietary guidelines or other nonmassage therapies (C), effective relief of constipation symptoms and associated symptoms (O), and randomized, controlled trials (RCTs) (S). According to PICOS components, we developed the searching strategy of this systematic review. The databases were searched with a combination of medical subject heading (MeSH) terms and text words for target population and interventions using massage. The detailed retrieval strategy is in the appendix file. [file 8934751.f1.docx]

# Evaluating the Efficacy of Massage Intervention for the Treatment of Post-Stroke Constipation: A Meta-Analysis

Qiu-Shuang Wang, Ya Liu, Xiang-Ni Zou, Yan-Ling Ma, and Gen-Li Liu

**Supplementary Material: Search strategy**

**PubMed**

[(((("Constipation"[Mesh]) OR ((((gastrointestinal constipation) OR bowel disorder) OR colonic inertia) OR dyschezia))) AND (("Massage"[Mesh]) OR (((((((OR oil massage) OR therapeutic touch) OR manual therapy) OR massotherap*) OR friction) OR trager) OR tactile-kinesthetic))) AND (("Stroke"[Mesh]) OR ((((((((apoplexy) OR cerebrovascular Accident*) OR cerebrovascular) OR apoplexy cerebrovascular stroke) OR brain vascular accident) OR cerebral stroke) OR acute stroke) OR acute cerebrovascular accident))](https://www.ncbi.nlm.nih.gov/pubmed?term=((((%22Constipation%22%5bMesh%5d)%20OR%20((((gastrointestinal%20constipation)%20OR%20bowel%20disorder)%20OR%20colonic%20inertia)%20OR%20dyschezia)))%20AND%20((%22Massage%22%5bMesh%5d)%20OR%20((((((((snead)%20OR%20oil%20massage)%20OR%20therapeutic%20touch)%20OR%20manual%20therapy)%20OR%20massotherap*)%20OR%20friction)%20OR%20trager)%20OR%20tactile-kinesthetic)))%20AND%20((%22Stroke%22%5bMesh%5d)%20OR%20((((((((apoplexy)%20OR%20cerebrovascular%20Accident*)%20OR%20cerebrovascular)%20OR%20apoplexy%20cerebrovascular%20stroke)%20OR%20brain%20vascular%20accident)%20OR%20cerebral%20stroke)%20OR%20acute%20stroke)%20OR%20acute%20cerebrovascular%20accident))&cmd=correctspelling)

**Database for Chinese Technical Periodicals (VIP)**

KY=（中风OR缺血性脑卒中OR高血压性脑出血OR急性期脑梗死OR脑血管疾病OR急性缺血性脑中风OR缺血性脑血管病OR脑血栓缺血性脑卒中OR急性缺血性中风病OR脑梗死) and KY=（便秘OR大便干燥）and KY=（按摩OR按摩疗法OR 推拿治疗OR推拿疗法OR推拿）

**China National Knowledge Infrastructure**

(SU=中风OR SU=脑卒中OR SU=脑梗死OR SU=脑栓塞OR SU=脑出血OR SU=脑血栓OR SU=蛛网膜下腔出血) AND (TI =干预OR TI =控制OR TI=管理) AND (SU=按OR SU=揉捏 OR SU=推拿 OR SU=按摩疗法OR SU=推拿疗法) AND (SU=便秘OR SU=大便困难OR SU=大便干燥OR SU=腹胀OR SU=腹痛OR SU=结肠无力) AND (SU=随机 OR FT=随机)

**Web of Science**

# 1 TS = (Stroke OR Apoplexy OR Cerebrovascular Accident* OR Cerebrovascular OR Apoplexy Cerebrovascular Stroke OR brain vascular accident OR Cerebral Stroke OR Acute Stroke OR Acute Cerebrovascular Accident)

#2 TS = (Constipation OR gastrointestinal constipation OR bowel disorder OR Colonic Inertia OR Dyschezia)

#3 TS = (Massage OR knead OR oil massage OR therapeutic touch OR manual therapy OR massotherap* OR friction OR tactile-kinesthetic)

#4 #3 AND #2 AND #1

**Cochrane library**

(Stroke [MeSH Terms] OR Apoplexy OR Cerebrovascular Accident* OR Cerebrovascular OR Apoplexy Cerebrovascular Stroke OR brain vascular accident OR Cerebral Stroke OR Acute Stroke OR Acute Cerebrovascular Accident OR Acute Stroke OR Cerebral Stroke) AND (intervention OR control) AND (Constipation [MeSH Terms] OR bowel disorder OR Colonic Inertia) AND Massage [MeSH Terms] OR knead OR oil massage OR therapeutic touch OR manual therapy OR massotherap* OR friction OR tactile-kinesthetic in Title, Abstract, Keywords from 1993 to 2019 in Trials

**EMBASE**

#1 stroke OR brain infarction OR stroke, lacunar

#2 intervention OR program* OR control OR management

#3 abdominal pain OR constipation

#4 massage OR manual lymphatic drainage OR naturopathy

#5 Limits: full text, 1993.1-2019.5, human

#6 #1and #2 and #3 and #4and #5

**Chinese Biomedical Literature Database**

#1 卒中[MeSH Terms] OR中风OR脑卒中OR脑中风OR脑血管意外OR脑血管中风OR脑血管意外

#2 干预 OR 治疗

#3 按摩[MeSH Terms] OR推拿OR掌拨OR捏脊OR气功推拿

#4 便秘 [MeSH Terms]

#5 Limits: full text, 1993.1-2019.5, human

#6 #1and #2 and #3 and #4

**Wanfang Database**

Title = ((“中风” OR “脑梗死” OR “脑卒中”)AND (“干预” OR “控制”) AND (“便秘”) AND (“按摩”)) or Abstract =((“中风” OR “脑梗死” OR “脑卒中”) AND (“干预” OR “控制”) AND (“便秘”) AND (“按摩”))or Keywords = ((“中风” OR “脑梗死” OR “脑卒中”) AND (“干预” OR “控制”) AND (“便秘”) AND (“按摩”))
